# Magnetic Communication Using High-Sensitivity Magnetic Field Detectors

**DOI:** 10.3390/s19153415

**Published:** 2019-08-04

**Authors:** Maurice Hott, Peter A. Hoeher, Sebastian F. Reinecke

**Affiliations:** 1 Faculty of Engineering, Kiel University, 24143 Kiel, Germany; 2 Helmholtz-Zentrum Dresden-Rossendorf (HZDR), 01328 Dresden, Germany

**Keywords:** wireless communication, magnetic induction communication, mobile sensing systems, magnetic sensors, RF-challenging environments

## Abstract

In this article, an innovative approach for magnetic data communication is presented. For this purpose, the receiver coil of a conventional magneto-inductive communication system is replaced by a high-sensitivity wideband magnetic field sensor. The results show decisive advantages offered by sensitive magnetic field sensors, including a higher communication range for small receiver units. This approach supports numerous mobile applications where receiver size is limited, possibly in conjunction with multiple detectors. Numerical results are supported by a prototype implementation employing an anisotropic magneto-resistive sensor.

## 1. Introduction

**Figure 1:**
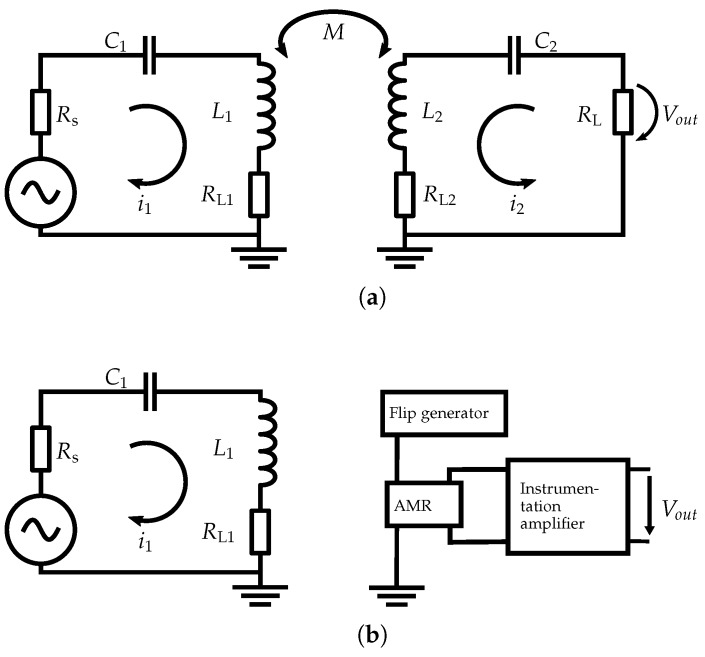
Conventional coil-to-coil topology (**a**) and coil-to-AMR topology under investigation (**b**).

Magneto-inductive (MI) communication is of growing interest in communication environments, where radio, optical wireless, acoustical, and molecular communications are challenging or not possible [1]. Examples include underwater communication [2,3], underground communication [4,5], process engineering such as communication in chemical reaction tanks, and medical applications [6]. MI communication is characterized by low propagation delay and small susceptibility to the surrounding environment [7]. Furthermore, in the near field there are no issues such as multipath propagation and fading. However, near-field MI communication has inherent restrictions. Due to a high attenuation of 60 dB per decade only short distances are supportable with reasonable data rates. According to the state-of-the-art, for communication purposes coils are used at the transmitter and receiver sides. The coils can be described as antennas. Inductive coupling enables a communication link between the transmitter and the receiver. The transmitter coil induces a modulated electrical current in the receiver coil. The voltage at the clamps of the receiver coil is evaluated as the received signal (Figure 1a). To reach reasonable distances, the transmitter and the receiver coil should operate in a tuned resonant circuit. This principle generates a significant gain for signal frequencies around the resonant frequency, but reduces the possible bandwidth depending on the quality factor of the resonant circuit. Since the communication range is limited by the radius of the transmitter and the receiver coils, mobile applications such as autonomous underwater vehicles (AUVs) or sensor networks are not efficient, if small coils are required at both ends.

In this publication, an innovative MI communication system approach is introduced: the receiver coil of a conventional MI communication system is replaced by a high-sensitivity magnetic field detector, in our prototype an anisotropic magneto-resistive (AMR) detector (Figure 1b). These detectors enable small and lightweight receiver units suitable for mobile applications. This proposal promises many benefits that could lead to cheap and reliable system design, among other advantages. Precise magnetic detectors have been applied for localization and navigation purposes, see for example [8,9,10]. To the authors best knowledge, however, high-sensitivity magnetic field detectors have not been studied in the area of data communication. In this sense, our contribution is original.

The paper is structured as follows. First, characteristics of magnetic field detectors are assessed in the context of MI communication. Afterwards, the sensitivity of coil-to-coil and coil-to-AMR communication is analyzed and compared. Finally, the results are summarized, and an outlook is given.

## 2. Characteristics of Magnetic Field Detectors

Presently, various magnetic sensors are commercially available. Most of the sensors measure the magnetic flux density of the magnetic field based on different sensing principles. For certain measurement tasks certain types of magnetic field sensors have been established. For example, giant magneto-resistive (GMR) sensors are used in reading heads of hard disks, whereas superconducting quantum interference devices (SQUIDs) are applied for measuring weak biomagnetic fields. In this section, the most common sensors are characterized regarding their suitability as a wideband receiver in a magnetic communication system. The crucial characteristics with respect to mobile communication purposes are summarized in Table 1.

- SQUIDs are ultra-sensitive magnetic field sensors [11]. The sensors can measure magnetic flux densities on the order of fT (for example brain activity tracking by magnetoencephalography). To maintain superconductivity, SQUIDs need to operate within a few degrees of absolute zero. This is ensured by cooling the devices with liquid helium, which is certainly not possible in mobile applications.
- The optical pumping resonance method exhibits very high sensitivity at low frequencies, but the sensor is large and is only able to detect the scalar (i.e., total) value of a magnetic field. The major problem regarding communication purposes is that the sensitivity of these magnetometers decreases rapidly for frequencies above 10 Hz [12]. This prevents communication at reasonable data rates.
- Hall effect sensors are among the most popular magnetic field sensors. The sensors are very cheap and are mainly used as proximity indicators. They cannot be used for navigation purposes due to their low sensitivity [9]. Most properties of Hall effect sensors are suitable for magnetic communication systems. However, the high detection threshold results in very short communication ranges.
- Fluxgate sensors can detect magnetic fields on the order of pT. Due to the small size and battery power supply, fluxgate magnetometers are qualified for mobile applications. The achievable data rate is limited by the upper frequency of a fluxgate sensor, which is about 10 kHz [12].
- Another well-known type of detector is magneto-resistive sensors. GMR and AMR sensors are implemented today in almost every smartphone, e.g. for compass functionality. Both sensor types require ultra-low power consumption and are cheap. The main advantage is the high bandwidth of up to 1 MHz and beyond. Due to the lower detection limit of AMR sensors, this method is favorable for communication systems.

Beside the features mentioned in Table 1, depending on the application other characteristics should be addressed, such as linearity, demagnetization caused by high field strengths, and directivity of the magnetic field.

In the following, the receiver coil of a conventional MI communication system (Figure 1a) and the AMR sensor as an example for the proposed topology (Figure 1b) are described in more detail.

### 2.1. Receiver Coil (Induction)

Coil-based magnetometers employ the principle of MI. This implies that a change of the magnetic flux density through a coil leads to a voltage difference between the clamps of the coil. The voltage difference is proportional to the alternating magnitude of the magnetic flux density. The magnetic flux density through a coil changes if it is exposed to a time-varying magnetic field. Based on the sensitivity analysis made in [12], the dynamic range of a coil-based detector is large compared to other magnetic sensors. By inserting a high-permeability coil core material and optimizing the size of the coil, magnetic flux densities *B* as weak as $B_{\min}=20$ fT can be measured [12].

### 2.2. AMR Detector

The AMR effect has been discovered by William Thomsen in Glasgow in 1857 [13]. He found out that single-domain ferromagnetic thin-films have a two per cent higher resistance in the direction of magnetization than in the orthogonal direction. Between these orientations, the resistance of an AMR thin filmstrip in a magnetic field can be described as a function of angle. In an AMR sensor, this effect is used for the detection and measurement of magnetic field magnitudes and directions. Frequently, the single-domain thin filmstrip is implemented in a Wheatstone bridge. The measured voltage difference depends on the magnitude and direction of the magnetic field [11].

**Figure 2:**
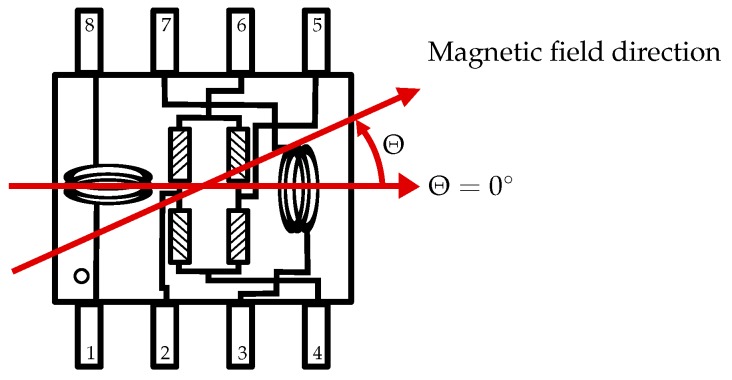
AMR sensor schema referring to [14].

In our prototype implementation, a Sensitec AFF755 low noise AMR sensor in a SO8-housing is used (Figure 2) [14]. The typical sensitivity is specified as 15 $\frac{\mathrm{mV}/V}{\mathrm{kA}/m}$. The noise level is given as 10 $\frac{\mathrm{nV}}{\sqrt{\mathrm{Hz}}}$ for frequencies above 100 Hz up to the cut-off frequency, which is about 1 MHz. For an assumed bandwidth of 999.9 kHz this corresponds to a noise amplitude spectral density of N≈9.5 µV without considering the noise of the instrumentation amplifier. The resulting detection threshold of the magnetic flux density of this AMR sensor is given as $B_{\min}\approx 93$ nT if the full bandwidth is exploited. Accordingly, for 100 kHz bandwidth $B_{\min}\approx 29$ nT.

A flip coil is implemented between pin 1 and 8 for offset correction and detection of weak magnetic fields [15]. If a sufficiently high electrical current flows through the integrated flip coil for a short time, a magnetic pulse is generated. In practice, pulses with alternating signs are generated. After every pulse the current direction changes, thus the generated magnetic field is directed opposite to the actual ferromagnetic thin-film-magnetization. This bipolar magnetic pulse flips the magnetization of the ferromagnetic thin film between two stable states. Every magnetic pulse forces a new alignment of the internal magnetic domains. This principle ensures the full sensitivity of the AMR sensor for the detection of weak magnetic fields. The flipping of the magnetization direction can also be used for offset correction of the AMR sensor.

## 3. Sensitivity Analysis

In this section, theoretical investigations and the corresponding numerical results are presented, based on measurements of our prototype implementation. For this purpose, the near-field far-field boundary is elaborated and the equation for transmission range is derived for coil-to-coil and coil-to-AMR communication. Then, the receiver circuit is characterized. Subsequently, coil-to-coil and coil-to-AMR communications are compared regarding different aspects.

### 3.1. Near-Field Far-Field Boundary

In antenna engineering, the near-field and the far-field are spatial regions with different characteristics. In the region close to an antenna, i.e., in the near field, there is no electro-magnetic (EM) radiation. In the far-field region, a combination of electrical and magnetic waves, known as EM wave, is radiated. For a coil system, the distance *d* at which the near-field passes into the far-field is conventionally defined by the frequency *f* of the transmitted signal and the speed of light in the medium $c_{\mathrm{medium}}$ [16]:

(1) $$d=\frac{c_{\mathrm{medium}}}{2\pi f}.$$

This equation is valid for all setups where the size of the antenna is much smaller than the wavelength. The maximum transmission range of a near-field MI communication system is limited by the corresponding near-field far-field boundary. For example, the near-field far-field boundary in air for $c_{\mathrm{air}}=\;3\times 10^{8}$ m/s and f=100 kHz is given as $d_{100\mathrm{kHz}}\approx 477$ m.

### 3.2. Communication Range for Lossless Transmission Mediums

An essential property for communication systems is the maximal transmission distance. In this section, the influence of different crucial parameters on the communication range is theoretically analyzed for coil-to-coil and coil-to-AMR communication. The system parameters used in the numerical results throughout this paper are listed in Table 2. The transmission medium is assumed to be lossless.

#### 3.2.1. Derivation of the Coil-to-Coil Transmission Range

In conventional MI communication systems, the properties of transmitter and receiver coil and the coupling between the coils have an influence on the transmission range. The basis for the calculation of the communication characteristics is the Agbinya-Masihour model [16]. The MI link budget can be expressed by the following equation:

(2) $$d_{\mathrm{cc}}^{6}{\left(1+\frac{r_{T}^{2}}{d_{\mathrm{cc}}^{2}}\right)}^{3}=\frac{P_{T}Q_{T}Q_{R}\eta_{T}\eta_{R}\mu_{r,T}\mu_{r,R}r_{T}^{3}r_{R}^{3}\pi^{2}}{P_{R}},$$

where $d_{\mathrm{cc}}$ is the transmission range, $P_{T}$ the transmitter power, $P_{R}$ the receiver sensitivity, $Q_{T}=\frac{2\pi f_{0}L}{R_{L1}+R_{S}}$ the transmitter quality factor, $Q_{R}=\frac{2\pi f_{0}L}{R_{L2}+R_{L}}$ the receiver quality factor, $\eta_{T}=\frac{R_{S}}{R_{S}+R_{L1}}$ the transmitter coil efficiency, $\eta_{R}=\frac{R_{L}}{R_{L}+R_{L2}}$ the receiver coil efficiency, $\mu_{r,T}$ the relative permeability of the core material of the transmitter coil, $\mu_{r,R}$ the relative permeability of the core material of the receiver coil, $r_{T}$ the transmitter coil radius, and $r_{R}$ the receiver coil radius. Since distance depends on $Q_{T}\cdot \eta_{T}$ and $Q_{R}\cdot \eta_{R}$, where $Q_{T}\cdot \eta_{T}$ is fixed while $Q_{R}\cdot \eta_{R}$ is a function of load resistance $R_{L}$, in Table 2 we have optimized $R_{L}$ in order to approach maximum distance. Equation (2) can be separated in a term with a significant influence on the determination of the distance and a so-called correction term with a very small effect on $d_{\mathrm{cc}}$:

(3) $$d_{\mathrm{cc}}=d^{\prime }\cdot \Delta d,$$

where

(4) $$d^{\prime }=\sqrt[{6}]{\frac{P_{T}Q_{T}Q_{R}\eta_{T}\eta_{R}\mu_{r,T}\mu_{r,R}r_{T}^{3}r_{R}^{3}\pi^{2}}{P_{R}}},$$

and the correction term is

(5) $$\Delta d=\frac{1}{\sqrt{1+\frac{r_{T}^{2}}{d_{\mathrm{cc}}^{\prime 2}}}}.$$

If the communication distance is large compared to the transmitter coil radius, the following approximation holds:

(6) $$\frac{1}{\sqrt{1+\frac{r_{T}^{2}}{d_{\mathrm{cc}}^{\prime 2}}}}\approx 1,\mathrm{for}r_{T}\ll d_{\mathrm{cc}}.$$

This simplifies the link budget, so that the transmission range $d_{\mathrm{cc}}$ can be approximated as $d_{\mathrm{cc}}\approx d^{\prime }$.

#### 3.2.2. Derivation of the Coil-to-AMR Transmission Range

If an AMR detector is used, the maximum transmission distance depends on the magnetic flux density *B* at the receiver side [17]:

(7) $$B=\frac{\mu_{0}\mu_{r,T}NIr_{T}^{2}}{2d_{\mathrm{cAMR}}^{3}},$$

where $\mu_{0}=4\pi \times 10^{-7}$ is the magnetic field constant, *N* the number of turns of the transmitter coil, and *I* the electrical current through the transmitter coil. Accordingly, the transmission range $d_{\mathrm{cAMR}}$ is

(8) $$d_{\mathrm{cAMR}}=\sqrt[{3}]{\frac{\mu_{0}\mu_{r,T}NIr_{T}^{2}}{2B}}.$$

If *B* is replaced by the detection threshold of the magnetic sensor, $B_{\min}$, the maximum communication range is obtained.

#### 3.2.3. Characterization of the Detection Threshold for the Prototype Implementation

Equation (8) proves that the detection threshold $B_{\min}$ should be small to reach high communication distances. The detection threshold of the receiver prototype implementation is the minimum magnetic flux density which can be measured, and results from the AMR sensor sensitivity and the noise level of the receiver circuit. For magnetic flux densities below that voltage, the generated magnetic field of the wanted signal is smaller than the noise voltage level of the receiver circuit. In the following, the sensitivity and the noise level of our prototype system are characterized, and the resulting detection threshold is calculated.

**Figure 3:**
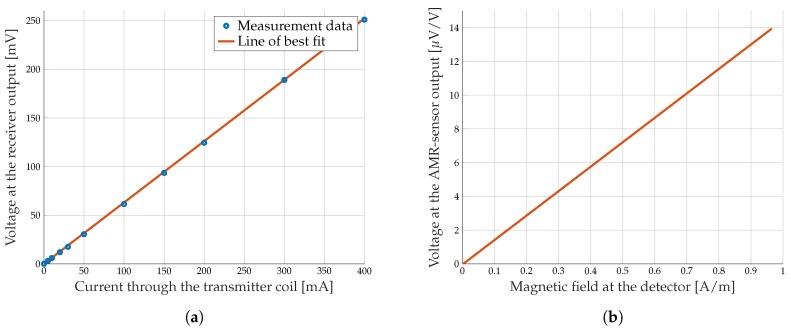
Measured voltage at the receiver output for different current through the transmitter coil (**a**) and the AMR sensor sensitivity curve (**b**).

To verify the given AMR sensitivity, the receiver is placed at a distance of *d* = 10.1 cm in boresight direction regarding the transmitter coil. Then, the output voltage of the receiver is measured for different magnetic flux densities. The magnetic flux density, which is present at the AMR sensor, is a function of the coil properties, the current through the transmitter coil, and the distance between transmitter and receiver. Figure 3a shows the voltage at the output of the receiver over the current through the transmitter coil for the fixed coil properties specified in Table 2. In addition to the measurement points, the resulting line of best fit is plotted.

To get the AMR sensitivity, the AMR output voltage before signal amplifying must be calculated and normalized to 1 V input voltage. Therefore, the voltage at the receiver output is divided by the amplifier gain (G = 2000) and the AMR sensor input voltage. Figure 3b shows the calculated voltage over the magnetic field, which is present at the AMR sensor. The AMR sensitivity can be read easily from the slope of the function. This results in a sensitivity of 14.33 $\frac{\mathrm{mV}/V}{\mathrm{kA}/m}$ for the embedded AMR sensor in our prototype receiver circuit. The value is very close to the specification of 15 $\frac{\mathrm{mV}/V}{\mathrm{kA}/m}$ which is given by the datasheet.

**Figure 4:**
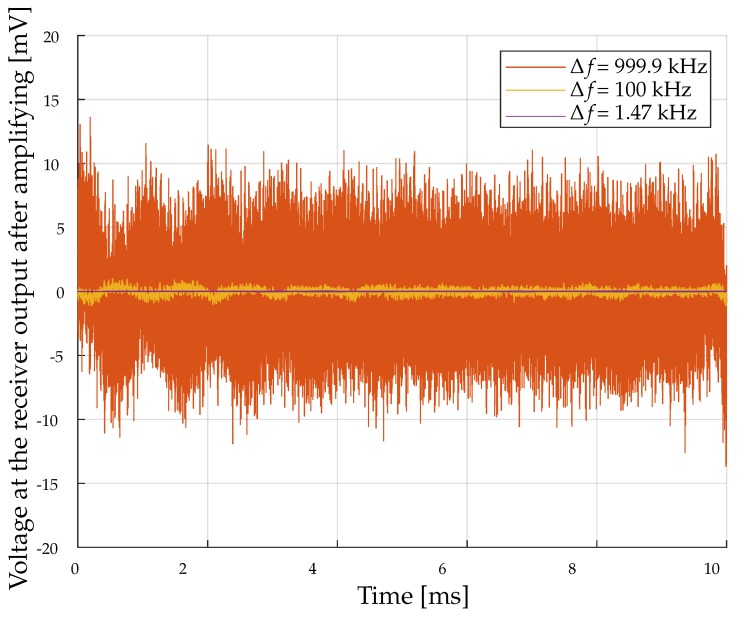
Measured noise signal for different bandwidths.

The second important parameter that affects the detection threshold, is the noise level of the receiver. The magnetic field sensor in our prototype system can work at frequencies from 0 Hz up to 1 MHz. The main components that contribute to the total noise are the AMR sensor with 10 $\frac{\mathrm{nV}}{\sqrt{\mathrm{Hz}}}$ and the noise of the amplifier with 1.5 $\frac{\mathrm{nV}}{\sqrt{\mathrm{Hz}}}$ for frequencies beyond 100 Hz [14,18]. For frequencies below 100 Hz, the total noise voltage is much higher so that these frequencies should be avoided for a small detection threshold. In this experiment, the total noise for different bandwidths is investigated. The chosen bandwidths are 1.47 kHz, 100 kHz and 999.9 kHz. 1.47 kHz, for direct comparison, exactly corresponds to the 3 dB bandwidth of the coil-to-coil communication for the prototype system parameters. Figure 4 shows the noise voltage over a period of 10 ms for the measured bandwidths.

Based on the measured receiver sensitivity and the noise voltages for different bandwidths the corresponding detection thresholds can be determined. The results are compared with the detection thresholds calculated with the theoretical values given in the datasheets. For the bandwidth Δf=1.47 kHz, the detection threshold is $B_{\min,1.47\;\mathrm{kHz}}\approx$ 5 nT (theoretical value: 4 nT), for Δf=100 kHz the detection threshold is $B_{\min,100\;\mathrm{kHz}}\approx$ 39 nT (theoretical value: 35 nT) and for Δf=999.9 kHz the detection threshold is $B_{\min,999.9\;\mathrm{kHz}}\approx$ 155 nT (theoretical value: 112 nT). It is clearly evident that the detection thresholds based on the measurement data are in the same order of magnitude as the calculated detection threshold based on the values given by the datasheet. For the following numerical calculations, the minimum detectable magnetic flux densities for the three different bandwidths of the coil-to-AMR communication are compared with the coil-to-coil communication.

#### 3.2.4. Transmission Range for Different Coil Radii

Equations (3) and (8) show that the coil radii have a direct influence on the maximum transmission range. In the case of coil-to-coil transmission, the radii of the transmitter coil and the receiver coil can be adapted. For coil-to-AMR communication, only the coil radius at the transmitter side is scalable.

**Figure 5:**
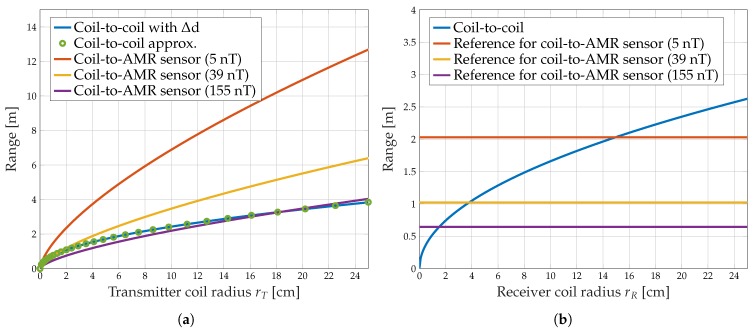
Transmission range for coil-to-coil and coil-to-AMR communication depending on the transmitter coil radius (**a**) and receiver coil radius (**b**). Parameters are taken from Table 2.

In the following, the influence of the transmitter and receiver coil radii on the coil-to-coil and the transmitter radius on the coil-to-AMR communication is investigated. Let us start with air coils, i.e., $\mu_{r}\approx 1$. Figure 5a shows the transmission range as a function of the transmitter coil radius $r_{T}$ for coil-to-coil and coil-to-AMR communications. As can be seen, the maximum transmission distance increases strictly monotonically with the transmitter coil radius for both communication techniques. It is remarkable that the transmitter coil radius has a stronger influence for coil-to-AMR than for coil-to-coil communications. Furthermore, the figure indicates that the correction term (5) is of negligible influence.

In the next investigation, the receiver side of both communication approaches is examined. For this purpose, the receiver coil radius for coil-to-coil transmission is varied. Figure 5b shows the range of coil-to-coil communication as a function of the receiver coil radius. As expected, the transmission range of the coil-to-coil system increases with the receiver coil radius. The maximum transmission distance for the coil-to-AMR system is determined by $B_{\min}$ of the magnetic field detector and is shown in the figure as reference values for the three different detection thresholds. For the given system parameters and e.g., $B_{\min}=5$ nT, the range of coil-to-AMR communication is outperformed by coil-to-coil communication beyond a receiver coil radius of $r_{R}\approx 15$ cm.

The results indicate that the range for coil-to-coil communication is severely restricted when small receiver coil radii are required. This is necessary for mobile applications such as AUVs and sensor networks. For these requirements, coil-to-AMR communication can serve much longer distances.

#### 3.2.5. Transmission Range for Different Core Materials

Equation (7) shows that the resulting magnitude of the magnetic flux density generated by a transmitter coil directly depends on the magnetic permeability of the core material. This can be explained by the fact that a high-permeability core acts as a flux concentrator inside a coil [19]. According to the Agbinya-Masihpour MI link budget (2), a high-permeability coil core can further be used at the receiver side of a coil-to-coil communication system to enhance the maximum communication distance.

Subsequently, the influence of the permeability of the coil core material on the transmission range is examined for both communication techniques. For coil-to-coil communication, the transmitter and receiver coil core permeability are adapted simultaneously. For coil-to-AMR communication, only the transmitter coil core permeability can be changed.

**Figure 6:**
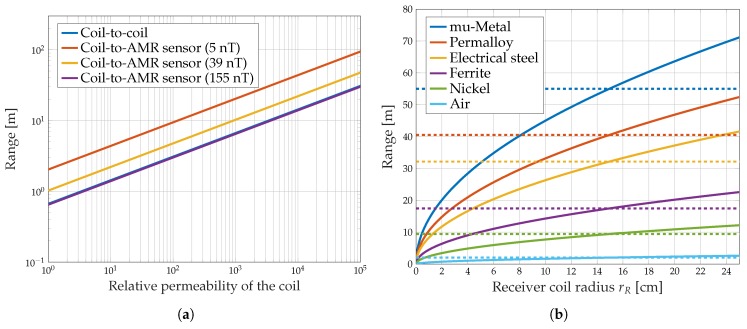
Transmission range for coil-to-coil and coil-to-AMR communication depending on the permeability (**a**) and the receiver coil radius for different core materials (**b**). Parameters are taken from Table 2 and Table 3. The dashed lines are for coil-to-AMR transmission.

Figure 6a presents the effect of the relative core permeability on the maximum transmission range for coil-to-coil and coil-to-AMR transmission. For both communication techniques, the range increases with the permeability of the coil core. Due to the same slope of the curves in log-log scale, they must follow the same power function.

Figure 6b shows the maximum coil-to-coil transmission range versus the receiver coil radius for materials with different permeabilities (Table 3). The corresponding coil-to-coil transmission range is compared to the coil-to-AMR communication reference range (dashed line) with different transmitter coil materials and a receiver detection bound of $B_{\min}=5$ nT for a bandwidth of 1.47 kHz. The maximum transmission distance for coil-to-coil and coil-to-AMR transmission is strictly monotonically increasing with the permeability of the core material by the same factor for both techniques. If for example a ferrite core is used, the maximal distance increases by a factor of 8.6. These numerical results reveal for a variety of different core materials that the influence of the core permeability for coil-to-coil communication and coil-to-AMR communication is the same. The advantage of coil-to-AMR communication is that the change of the transmitter coil core material leads to the same result as changing transmitter and receiver core material in a coil-to-coil communication system. Consequently, increasing the communication range can be realized for coil-to-AMR transmission with less effort than for coil-to-coil transmission.

However, the enhancement of the possible communication distance by means of increasing the permeability of the core material is limited by a few parameters. Primarily, there are no materials available that exhibit arbitrary high permeabilities. Additionally, the permeability of a material depends on the frequency, so that extremely high permeabilities for high frequencies usually cannot be achieved. Moreover, the saturation flux density $B_{s}$ of a core material is a constraint (Table 3). This results in the challenge to choose a core material with a uniformly high permeability for the frequency range which is used for communication and even a sufficiently high saturation flux density. A further restriction of the maximum applicable permeability of the coil core material arises due to the associated increase of the inductance *L* according to L∼μ. The inductance *L* of a coil has a limiting effect on the resonant frequency

(9) $$f_{0}=\frac{1}{2\pi \sqrt{LC}},$$

because the capacity *C* of the resonant circuit is lower bounded by parasitic capacitances.

#### 3.2.6. Coil-to-Detector Transmission Range for Different Detection Thresholds

**Figure 7:**
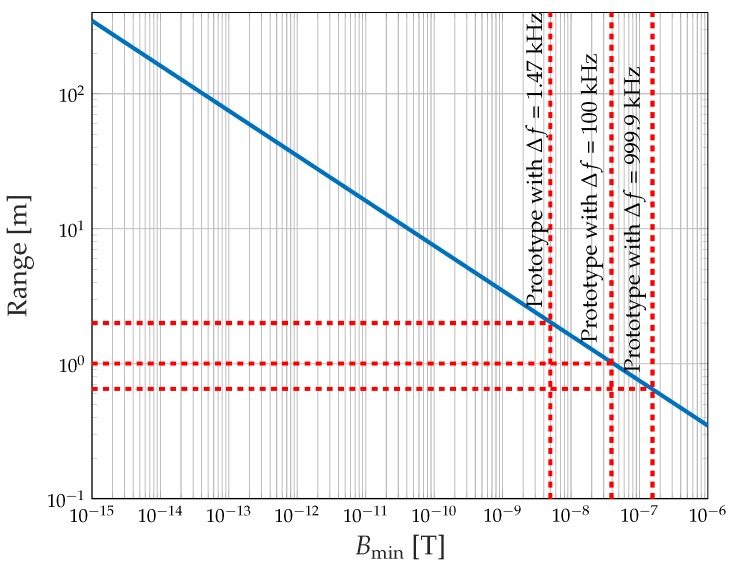
Transmission range as a function of the magnetic field detection limit of the detector. Parameters are taken from Table 2. The maximum transmission distance based on the prototype implementation detection threshold is marked for three different bandwidths. The transmission range can be increased by using a more sensitive type of detector.

Now, the influence of the sensor’s detection limit, $B_{\min}$, on the maximum transmission distance is investigated. Figure 7 shows the transmission range for coil-to-detector communication as a function of $B_{\min}$. The range is strictly monotonically increasing for increasing sensitivity. Therefore, it is recommendable to use the most sensitive sensors that are available and applicable for the communication scenario of interest. This allows much longer transmission distances for the same remaining system parameters. In the figure, the maximum communication distance for the three investigated bandwidths is marked. For Δf=1.47 kHz the maximum distance is 2 m, for Δf=100 kHz the communication range is 1 m, and if the maximum bandwidth Δf=999.9 kHz exploited the range results in 0.65 m.

## 4. Conclusions and Outlook

In this contribution, an innovative approach for magnetic communication is considered. The key idea is to employ a high-sensitivity wideband magnetic field sensor at the receiver side. Several advantages have been indicated compared to conventional MI coil-to-coil communication, because a different measurement principle is used compared to a receiver coil. In most of our numerical results an AMR sensor is assumed. AMR receivers are extremely small, so that they can be considered to be a point sink. Given the same form factor, they are much more sensitive than coils. This enables longer transmitting distances for mobile applications, where small receiver sizes are necessary. The frequency characteristic of AMR sensors is almost flat below the cut-off frequency. This feature together with the small size is of great interest for multiple-input single-output (MISO) system approaches. Vice versa, single-input multiple-output (SIMO) processing can be used for sensitivity improvements. Some AMR detectors include 3D functionality. This aspect is useful for joint communication and localization purposes. Possibly, the data rate can be enhanced by 3D processing. Moreover, a high permeability transmitter core material can be used for coil-to-AMR communication to extend the possible range drastically. Our investigation demonstrates that magnetic field communication with an AMR receiver is a promising technique for mobile applications in challenging environments. Since the effective transmission distance is a function of the sensitivity of the detectors, the fundamental observations of this paper can be exported to other measurement principles. For example, the AMR sensor used in our prototype receiver could be replaced by a fluxgate sensor.

## Figures and Tables

**Table 1:** Comparison of magnetic sensors regarding mobile communication purposes.

	SQUID	Optical Pumping	Hall	Fluxgate	GMR/TMR	AMR	Induction
Detection limit	fT	pT	μT	pT	nT	nT	fT-T
Power supply	Line	Line	Battery	Battery	Battery	Battery	Battery
Size	Medium	Medium	Ultra small	Small	Small	Ultra small	Ultra small-large
Measured value	Vectorial	Scalar	Vectorial	Vectorial	Vectorial	Vectorial	Vectorial
Sensor noise	Ultra-low	Ultra-low	High	Low	High	Medium	Low
Bandwidth	Small	Small	Medium	Medium	Medium	Large	Small
Saturation by Earth’s magnetic field	Yes	Yes	No	No	No	No	No
Operating temperature	Ultra-low	Normal	Normal	Normal	Normal	Normal	Normal
Cost	High	High	Low	Medium	Low	Low	Low
Suitability for mobile applications	No	No	Yes	Yes	Yes	Yes	Only small coils

**Table 2:** Prototype system parameters.

Meaning	Parameter	Value
Relative permeability of the core	$\mu_{r}$	1
Quality factor transmitter	$Q_{T}$	68
Quality factor receiver	$Q_{R}$	35
Efficiency transmitter	$\eta_{T}$	2.4%
Efficiency receiver	$\eta_{R}$	50%
Bandwidth transmitter	Δf	1.47 kHz
Coil radius	$r_{T}$, $r_{R}$	16 mm
Number of turns	*N*	20
Inductance	*L*	14.35 μH
Resonant frequency	$f_{0}$	100 kHz
Series resistance of power supply	$R_{S}$	3.2 mΩ
Load resistance	$R_{L}$	129 mΩ
Series resistance of coil	$R_{L1}$, $R_{L2}$	129 mΩ @ 100 kHz
Transmitting power	$P_{T}$	43.3 dBm
Receiver sensitivity	$P_{R}$	−42 dBm

**Table 3:** Magnetic permeability μ in Henries per meter, relative permeability $\mu_{r}$ and saturation flux density $B_{s}$ in tesla for different materials. For μ and $\mu_{r}$ the initial permeability of the respective core material is given in the table [16,19,20].

Material	μ	$\mu_{r}$	$B_{s}$
Air	$1.2566\times 10^{-6}$ H/m	≈1	no saturation
Nickel	$1.25\times 10^{-4}$ H/m	≈100	0.3–0.5 T
Ferrite	>$8\times 10^{-4}$ H/m	>640	0.3–0.5 T
Electrical steel	$5\times 10^{-3}$ H/m	≈4000	1.5–1.8 T
Permalloy	$10^{-2}$ H/m	≈8000	0.66–0.82 T
mu-Metal	≈$2.5\times 10^{-2}$ H/m	≈20,000	0.65–0.82 T

## References

[1] Akyildiz I.F., Vuran M.C. (2010). Wireless Sensor Networks.

[2] Domingo M.C. (2012). Magnetic Induction for Underwater Wireless Communication Networks. IEEE Trans. Antennas Propag..

[3] Akyildiz I.F., Wang P., Sun Z. (2015). Realizing underwater communication through magnetic induction. IEEE Commun. Mag..

[4] Sun Z., Akyildiz I.F. (2010). Magnetic Induction Communications for Wireless Underground Sensor Networks. IEEE Trans. Antennas Propag..

[5] Kisseleff S. (2017). Advances in Magnetic Induction Based Underground Communication Systems. Ph.D. Thesis.

[6] Masihpour M., Agbinya J.I. Cooperative relay in Near Field Magnetic Induction: A new technology for embedded medical communication systems. Proceedings of the 2010 Fifth International Conference on Broadband and Biomedical Communications.

[7] Agbinya J.I. (2011). Principles of Inductive Near Field Communications for Internet of Things.

[8] Carrella S., Kuncup I., Lutz K., König A. 3D-Localization of Low-Power Wireless Sensor Nodes Based on AMR-Sensors in Industrial and AmI Applications. Proceedings of the Sensoren und Messsysteme.

[9] Včelák J., Ripka P., Zikmund A. (2015). Precise magnetic sensors for navigation and prospection. J. Supercond. Nov. Magn..

[10] Reinecke S.F., Hampel U. (2016). Instrumented flow-following sensor particles with magnetic position detection and buoyancy control. J. Sens. Sens. Syst..

[11] Ripka P., Janosek M. (2010). Advances in Magnetic Field Sensors. IEEE Sens. J..

[12] Lenz J., Edelstein S. (2006). Magnetic sensors and their applications. IEEE Sens. J..

[13] Thomson W. (1856). On the Electro-Dynamic Qualities of Metals:–Effects of Magnetization on the Electric Conductivity of Nickel and of Iron. Proc. R. Soc. Lond..

[14] Sensitec (2017). AFF755B MagnetoResistive Field Sensor. https://www.sensitec.com/fileadmin/sensitec/Service_and_Support/Downloads/Data_Sheets/AFF700_800/SENSITEC_AFF755B_DSE_05.pdf.

[15] Nording F., Bischel W., Ludwig F., Schilling M. (2017). Offsetunterdrücktes AMR-Magnetometer mit 100 kHz Frequenzbandbreite. tm-Tech. Mess..

[16] Masihpour M. (2012). Cooperative Communication in Near Field Magnetic Induction Communication Systems. Ph.D. Thesis.

[17] Coey J.M. (2010). Magnetism and Magnetic Materials.

[18] Devices A. (2012). AD8428 Instrumentation Amplifier. https://www.analog.com/media/en/technical-documentation/data-sheets/AD8428.PDF.

[19] Tumanski S. (2016). Handbook of Magnetic Measurements.

[20] McLyman C.W.T. (2016). Transformer and Inductor Design Handbook.

